# Hypertension prevalence in the *All of Us* Research Program among groups traditionally underrepresented in medical research

**DOI:** 10.1038/s41598-021-92143-w

**Published:** 2021-06-22

**Authors:** Paulette D. Chandler, Cheryl R. Clark, Guohai Zhou, Nyia L. Noel, Confidence Achilike, Lizette Mendez, Andrea H. Ramirez, Roxana Loperena-Cortes, Kelsey Mayo, Elizabeth Cohn, Lucila Ohno-Machado, Eric Boerwinkle, Mine Cicek, Jun Qian, Sheri Schully, Francis Ratsimbazafy, Stephen Mockrin, Kelly Gebo, Julien J. Dedier, Shawn N. Murphy, Jordan W. Smoller, Elizabeth W. Karlson, the All of Us Research Program Investigators, Habibul Ahsan, Toluwalase Ajayi, Alvaro Alonso, Amit Arora, Briseis Aschebrook-Kilfoy, Sally L. Baxter, Dean Billheimer, Eugene R. Bleeker, Luca Bonomi, Olveen Carrasquillo, Qingxia Chen, Dave Chesla, Andrew Craver, Zubin Dastur, The PRIDE Study/PRIDEnet, John Ehiri, Mara M. Epstein, Xiaoke Feng, Annesa Flentje, Alliance Health Project, Lawrence Garber, Nicholas Giangreco, Yi Guo, Robert A. Hiatt, Kai Yin Ho, Joyce Ho, William Hogan, George Hripcsak, Carolyn Hunt, Rosario Isai, Xinzhuo Jiang, Christine C. Johnson, King Jordan, Christine LM. Joseph, Hooman Kamel, Jason H. Kames, Theresa H. Keegan, Karen Kim, Katherine K. Kim, Jihoon Kim, Paula King, Yann C. Klimentidis, Irving L. Kron, Tsung-Ting Kuo, Helen Lam, James P. Lash, Micah E. Lubensky, Mitchell R. Lunn, Yves A. Lussier, Jacob L. McCauley, Robert Meller, Deborah A. Meyers, Raul A. Montanex Valverde, Julia L. Moore Vogel, Shashwat D. Nagar, Kartnik Natarajan, Juno Obedin-Maliver, Paulina Paul, Pamela A. Pawloski, Cathryn Peltz-Rauchman, Priscilla Pemu, Fomessa T. Randal, Ana Rescate, Ana C. Ricardo, M. Elizabeth Ross, Brittney Roth-Manning, Madhi Saranadasa, Ning Shang, Emily G. Spencer, Cassie Springer, Alan Stevens, Vignesh Subbian, Amy Tang, Rhonda K. Trousdale, Jill Waalen, Stephen Waring, Chunhua Weng, Lisa White, Sonya White, Nathan E. Wineinger, Chen Yeh, Hsueh-Han Yeh, Paul Zakin, Yanhua Zhos, Stephan Zuchner, Robert Cronin, Alese Halvorson, Brian Ahmedani

**Affiliations:** 1 Brigham and Women′s Hospital, Harvard Medical School, Boston, MA 02115 USA; 2 Boston Medical Center, Boston University School of Medicine, Boston, MA USA; 3 Vanderbilt University Medical Center, Nashville, TN USA; 4 Hunter College, New York, NY USA; 5 University of California, San Diego, CA USA; 6 University of Texas Health Science Center School of Public Health, Houston, TX USA; 7 Mayo Clinic, Rochester, MN USA; 8 National Institutes of Health, Bethesda, MD USA; 9 Life Sciences Division, Leidos, Inc, Frederick, MD USA; 10 Johns Hopkins University, Baltimore, MD USA; 11 Research Information Science and Computing, Mass General Brigham, Boston, MA USA; 12 Center for Genomic Medicine, Massachusetts General Hospital, Boston, MA USA; 13 The University of Chicago, Chicago, IL USA; 14 Scripps Research, La Jolla, CA USA; 15 Emory University, Atlanta, GA USA; 16 University of Arizona, Tucson, AZ USA; 17 University of Miami, Miami, FL USA; 18 Vanderbilt University, Nashville, TN USA; 19 Spectrum Health, Temple, TX USA; 20 Stanford University, Stanford, CA USA; 21 PRIDEnet, Palo Alto, CA USA; 22 University of Massachusetts, Worcester, MA USA; 23 Columbia University, New York, NY USA; 24 University of Florida, Gainesville, FL USA; 25 University of California, San Francisco, San Francisco, CA USA; 26 Northwestern University, Chicago, IL USA; 27 Henry Ford Health Center, Detroit, MI USA; 28 Georgia Institute of Technology, Atlanta, GA USA; 29 Weill Cornell Medical Center, New York, NY USA; 30 University of California, Davis, Sacramento, CA USA; 31 Morehouse School of Medicine, Atlanta, GA USA; 32 Health Partners Institute, La Jolla, CA USA; 33 Asian Health Coalition, Chicago, IL USA; 34 University of Illinois At Chicago, Chicago, IL USA; 35 Baylor Scott & White Research Institute, Dallas, TX USA; 36 NYC Health and Hospital/Harlem, New York, NY USA; 37 Essential Institute of Rural Health, Duluth, MN USA

**Keywords:** Computational biology and bioinformatics, Cardiology, Health care, Risk factors

## Abstract

The *All of Us* Research Program was designed to enable broad-based precision medicine research in a cohort of unprecedented scale and diversity. Hypertension (HTN) is a major public health concern. The validity of HTN data and definition of hypertension cases in the *All of Us (AoU)* Research Program for use in rule-based algorithms is unknown. In this cross-sectional, population-based study, we compare HTN prevalence in the *AoU* Research Program to HTN prevalence in the 2015–2016 National Health and Nutrition Examination Survey (NHANES). We used *AoU* baseline data from patient (age ≥ 18) measurements (PM), surveys, and electronic health record (EHR) blood pressure measurements. We retrospectively examined the prevalence of HTN in the EHR cohort using Systemized Nomenclature of Medicine (SNOMED) codes and blood pressure medications recorded in the EHR. We defined HTN as the participant having at least 2 HTN diagnosis/billing codes on separate dates in the EHR data AND at least one HTN medication. We calculated an age-standardized HTN prevalence according to the age distribution of the U.S. Census, using 3 groups (18–39, 40–59, and ≥ 60). Among the 185,770 participants enrolled in the *AoU* Cohort (mean age at enrollment = 51.2 years) available in a Researcher Workbench as of October 2019, EHR data was available for at least one SNOMED code from 112,805 participants, medications for 104,230 participants, and 103,490 participants had both medication and SNOMED data. The total number of persons with SNOMED codes on at least two distinct dates and at least one antihypertensive medication was 33,310 for a crude prevalence of HTN of 32.2%. *AoU* age-adjusted HTN prevalence was 27.9% using 3 groups compared to 29.6% in NHANES. The *AoU* cohort is a growing source of diverse longitudinal data to study hypertension nationwide and develop precision rule-based algorithms for use in hypertension treatment and prevention research. The prevalence of hypertension in this cohort is similar to that in prior population-based surveys.

## Introduction

Hypertension (HTN) is a major public health concern and remains a leading risk factor for stroke and cardiovascular disease^1–4^. The diagnosis and treatment of HTN is straightforward, but the lack of control is commonplace with about 40% of treated patients achieving blood pressure targets in the United States^5^. Precision rule-based algorithms as tools for the development of hypertension treatment and prevention strategies are a promising solution^6^; the incorporation of multi-dimensional data that include genetics, nutrition, environment, and other biomarkers expand the potential prevention and intervention targets. AoU allows communities to participate in data collection further enriching the available data. Our rationale for this study was to validate the definition of HTN^7^ in the new resource, the *All of Us (AoU)* Research Program using rule-based algorithms. The validity of this definition based on electronic health record (EHR) data in underrepresented populations is unknown.

The National Institutes of Health Precision Medicine Initiative of which, the *AoU* Research Program is a component, is a longitudinal cohort study based on asking participants to play an active role in collecting and sharing their unique health information including EHR for use in precision medicine studies^8^. The aim is to enroll over a million participants who represent the diversity of the United States.

*AoU* demonstration project teams were charged with replicating known associations from published literature to demonstrate the utility of the data and to test the Researcher Workbench interface prior to release. Our aim was to use published methods^7^ to replicate known differences in HTN prevalence in groups underrepresented in biomedical research (UBR) and illustrate variation in HTN prevalence in geographic regions of the U.S**.** We compared our results to the 2015–2016 National Health and Nutrition Examination Survey (NHANES) HTN prevalence results^9^. Our findings may inform the use of *AoU* data to develop rule-based algorithms based on EHR data for prevention and treatment of hypertension in clinical practice.

## Methods

### All of Us demonstration projects

The goals, recruitment methods and sites, and scientific rationale for *AoU* have been described previously^8^. Demonstration projects were designed to describe the cohort, replicate previous findings for validation, and avoid novel discovery in line with the program value to ensure equal access by researchers to the data. The work described here was proposed by Consortium members, reviewed and overseen by the program’s Science Committee, and was confirmed as meeting criteria for non-human subject research by the *AoU* Institutional Review Board. All methods were carried out in accordance with relevant guidelines and regulations. Informed consent was obtained from all the participants. All experimental protocols involving human participants were approved by Ethics committee/Institutional Review Board (IRB) of the *AoU* Institutional Review Board.

The initial release of data and tools used in this work was published recently^10^. Results reported are in compliance with the *AoU* Data and Statistics Dissemination Policy disallowing disclosure of group counts under 20. *AoU* enrollment started in May 2018 and currently enrolls participants 18 years of age or older from a network of more than 340 recruitment sites^11^. From October, 2019 to February, 2020, 38 demonstration projects were performed using the *AoU* Research Program Curated Data Set (CDR) on a secure server, utilizing a Researcher Workbench interface. The Research Workbench included 188,781 participants.

### All of Us research hub

This work was performed on data collected by the previously described *AoU* Research Program^8^ using the *AoU* Researcher Workbench, a cloud-based platform where approved researchers can access and analyze data. The data currently includes surveys, EHR data and physical measurements (PM). The details of the surveys are available in the Survey Explorer found in the Research Hub, a website designed to support researchers^12^. Participants could choose not to answer specific questions. PM recorded at enrollment include systolic and diastolic blood pressure, height, weight, heart rate, waist and hip measurement, wheelchair use, and current pregnancy status. EHR data was linked for those consented participants. All three datatypes (survey, PM, and EHR) are mapped to the Observational Health and Medicines Outcomes Partnership (OMOP) common data model v 5.2 maintained by the Observational Health and Data Sciences Initiative (OHDSI) collaborative. To protect participant privacy, a series of data transformations were applied. These included data suppression of codes with a high risk of identification such as military status; generalization of categories, including age, sex at birth, gender identity, sexual orientation, and race; and date shifting by a random (less than one year) number of days, implemented consistently across each participant record. Documentation on privacy implementation and creation of the CDR is available in the *AoU* Registered Tier CDR Data Dictionary^13^. The Researcher Workbench currently offers tools with a user interface (UI) built for selecting groups of participants (Cohort Builder), creating datasets for analysis (Dataset Builder), and Workspaces with Jupyter Notebooks (Notebooks) to analyze data. The Notebooks enable use of saved datasets and direct query using R and Python 3 programming languages^10^. We used R version 4.0.3 to perform the analyses. We used EXCEL to create figures to display the hypertension prevalence and 95% confidence intervals.

Participants completed informed consent, provided consent for sharing of electronic health record data with the Data and Research Center (DRC), and provided survey responses on demographics, health status and behaviors including cigarette smoking, alcohol use, and illicit drug use at baseline.

### Definition of HTN

We defined HTN using the published electronic Medical Records and Genomics Network (eMERGE) algorithm (https://phekb.org/phenotype/resistant-HTN) for a study of resistant HTN cases versus controls with treated HTN^14^. The eMERGE definition for HTN required individuals to have an outpatient measurement of systolic blood pressure greater than 140 or diastolic blood pressure greater than 90 prior to meeting medication criteria or International Classification of Diseases, Ninth Revision, Clinical Modification (ICD-9-CM) code of 401.* (essential HTN) or *International Classification of Diseases, 10th Revision, Clinical Modification* (ICD-10-CM) code of I10 code (essential HTN) at any time and at least one medication from the HTN medication classes. The eMERGE network has published evidence of the improved positive predictive value (PPV) of using 2 instances of diagnosis/billing codes for phenotype algorithms in EHR data^15^. Since we did not have complete data on systolic and diastolic blood pressures from EHR across all sites, we adapted the eMERGE definition to include at least 2 diagnosis/billing codes on separate dates in the EHR data AND at least one HTN medication. We defined the index date for newly diagnosed HTN cases by date of first HTN medication code. We defined age at index date for HTN cases. Females or males were identified as participants with female or male sex assigned at birth.

### Data collection from in-person study visit and EHRs

Study protocols at each site were used to measure data on blood pressure at in-person “Physical Measurement” (PM) visits. Clinical data on blood pressure collected for routine patient care and recording in participant EHRs were extracted and transformed into OMOP tables at each enrollment site. Data was transferred securely to the Data Research Center at Vanderbilt University. PM visit and EHR data were used to identify blood pressure measurements for each data source. Survey data were used to collect data on demographics, including sex and gender identity, income, education, race/ethnicity, age, and geography (U.S. state of residence).

### EHR data extraction

We extracted SNOMED codes for essential HTN, defined the first SNOMED code, and defined a second SNOMED code on distinct date. A participant was defined as having HTN if two distinct SNOMED codes for HTN were identified. For the 48,289 participants with the SNOMED code for essential HTN (59,621,000) on any date, we extracted each participant’s detailed dates of SNOMED code for essential HTN from the Researcher Workbench table ‘cb_search_all_events’. We found 39,779 participants the SNOMED code for essential HTN on at least two distinct dates.

### Extraction of medication treatment history for anti-hypertensive medications

We selected medications from the following six classes based on RxNorm codes in the Researcher Workbench: peripheral vasodilators, agents acting on the renin-angiotensin system, beta blocking agents, antihypertensives, calcium channel blockers, and diuretics. The Researcher Workbench table ‘concept_ancestor’ was used to extract all medications within the six medication classes.

### Statistical analysis

Participants that had at least one Systemized Nomenclature of Medicine (SNOMED) code for HTN in their EHR were considered for the analysis. SNOMED codes are a standardized term for medical conditions used by healthcare providers for uniformity in diagnostics, billing and documentation. After considering multiple potential definitions, we decided to use the EHR data (SNOMED codes for HTN on 2 distinct dates and at least one HTN medication) as the primary definition of HTN^14^. For the 48,289 participants with the SNOMED code for essential HTN (59,621,000) on any date, we extracted each participant’s detailed dates of SNOMED code for essential HTN from the Researcher Workbench table ‘cb_search_all_events’. We selected medications from the following six classes based on RxNorm codes in the Researcher Workbench: peripheral vasodilators, agents acting on the renin-angiotensin system, beta blocking agents, antihypertensives, calcium channel blockers, and diuretics. The Researcher Workbench table ‘concept_ancestor’ was used to extract all medications within the six medication classes. We excluded SNOMED essential HTN codes (59,621,000) recorded on the same date as SNOMED pregnancy codes (24,898,207), There were 13,481 pregnant participants based on SNOMED pregnancy codes (24,898,207) and 1,665 with HTN and SNOMED pregnancy codes on the same date.

We calculated crude, and age-adjusted prevalence of HTN standardized by age from US Census data as in Crim et al.^7^ Based on methods used in Crim et al. paper^7^, we classified age at date of enrollment (e.g. PPI date) into 3 groups: 18–39, 40–59, ≥ 60, 4 groups: 18–39, 40–59, 60–74, ≥ 75, and 5 groups: 18–49, 50–59, 60–69, 70–79, ≥ 80^7^. We calculated an age-standardized HTN prevalence according to the age distribution of the U.S. Census. The census population size at each age group is as of July 1, 2018 and based on https://www.census.gov/newsroom/press-kits/2019/detailed-estimates.htmlA . Age-standardization was performed for 3 groups: 18–39, 40–59, ≥ 60; 4 groups: 18–39, 40–59, 60–74, ≥ 75; and 5 groups: 18–49, 50–59, 60–69, 70–79, ≥ 80. Race/ethnicity was coded into 6 groups based on *AoU* race and ethnicity variables in the Researcher Workbench as Non-Hispanic White race, Non-Hispanic Black race, Non-Hispanic Asian race, more than one race, other race (included Native Hawaiian and Other Pacific Islander, Middle Eastern and North African) and Hispanic ethnicity. The confidence interval for hypertension prevalence was computed using the Normal approximation interval based on the central limit theorem. We also tested for difference in HTN prevalence for males versus females with a Chi-square test. Socioeconomic status (SES) was classified on the income and education variables as a binary variable with low SES defined as low income (≤ $25,000) OR low education (< high school degree or GED) vs. not low in either category. Individuals with missing values for education or income were included in the group high income/high education based on the assumption that individuals with income higher than $25,000 might be more likely to have missing values for income and education than individuals with income less than $25,000. We assessed the agreement between the income and education variables by looking at the percent overlap of high income and high education versus low income and low education. We tested for significance of the overlap with a Chi-square test. For education and income, we did sensitivity analyses for crude HTN stratified by the education and income variables: low education (< high school degree or GED) versus high education (above high school or GED) and low income (≤ $25,000) versus high income (> $25,000). We reported the frequency of missing values for education and income. Geographic division of the U.S. was based on 9 U.S. Census Geographic divisions (https://www.cdc.gov/nchs/products/databriefs/db289.htm.): Division 1—New England (Maine, Vermont, New Hampshire, Massachusetts, Connecticut, and Rhode Island); Division 2—Middle Atlantic (New Jersey, New York, and Pennsylvania); Division 3—East North Central (Wisconsin, Michigan, Ohio, Indiana, and Illinois); Division 4—West North Central (North Dakota, South Dakota, Nebraska, Kansas, Missouri, Iowa, and Minnesota); Division 5—South Atlantic (Maryland, Delaware, West Virginia, Virginia, North Carolina, South Carolina, Georgia, Florida, and District of Columbia); Division 6—East South Central (Kentucky, Tennessee, Alabama, and Mississippi); Division 7—West South Central (Oklahoma, Arkansas, Texas, and Louisiana); Division 8—Mountain (Idaho, Montana, Wyoming, Colorado, Utah, Nevada, Arizona, and New Mexico); Division 9—Pacific (Washington, Oregon, California, Alaska, and Hawaii). West North Central Division (n = 1) and West South Central Division (n = 0) were excluded from analyses due to extremely low sample size. The Census division information for participants was derived from the PPI data.

## Results

Researcher Workbench EHR and medication data were available on 104,047 participants, SNOMED codes were available on 112,468 participants, and 103,270 participants had both medication and SNOMED data. Thus, 103,270 was the denominator for prevalence calculations. Sociodemographic differences for individuals with and without HTN are shown in Table 1.

**Table 1:** Sociodemographic Characteristics of the *All of Us* Research Program Electronic Health Record Dataset in January 2020 Among Participants with HTN and Without HTN^1^.

Variable	HTN (N = 33,267)	No HTN (N = 70,003)	P-value
**Race/ethnicity**			
Non-Hispanic white, %	49.8	53.1	< 0.001
Non-Hispanic black, %	28.1	17.5	< 0.001
Non-Hispanic Asian, %	1.6	2.9	< 0.001
More than one race, %	1.1	1.7	< 0.001
Other race, %	1.6	1.9	0.004
Hispanic, %	16.3	21.4	< 0.001
Unknown race, %	1.5	1.5	0.337
**Socioeconomic status**			
Low education or low income, %	49.7	43.4	< 0.001
Not low education/income, %	50.3	56.6	< 0.001
**Census division**			
New England, %	9.0	8.6	0.066
Middle Atlantic, %	29.4	25.7	< 0.001
East North Central, %	21.7	22.0	0.288
South Atlantic, %	0.0	0.0	1.000
East South Central, %	7.5	6.4	< 0.001
Mountain, %	8.4	5.1	< 0.001
Pacific, %	0.0	0.0	1.000
Unknown Division (geographic state info suppressed for privacy protection), %	9.8	17.1	< 0.001

^1^Numbers represent column percentage.

The total number of persons with SNOMED codes on at least two distinct dates and at least one antihypertensive medication was 33,310 for a crude prevalence of HTN of 32.2%. The crude prevalence was 7.7% among ages 18–39, 32% among ages 40–59, and 50.4% among ages ≥ 60 (Table 2). The census population size for each age group as of July 1, 2018 is shown in Table 2.

**Table 2:** Crude HTN Prevalence in *All of Us* Research Program with Age Distribution in *All of Us* Compared with the U.S. Census (2018)^1^.

Age Group	*All of Us* HTN crude prevalence, % (95% CI)	% of age group in *All of Us* cohort	% of age group in Census cohort
18–39	7.7 (7.4–8.0)	26.8	38.6
40–59	32.0 (31.5–32.4)	36.1	32.9
≥ 60	50.4 (49.9–50.9)	37.1	28.4

^1^https://www.census.gov/newsroom/press-kits/2019/detailed-estimates.html.

Crude HTN prevalence in *AoU* for each age group by gender is shown in Table 3

**Table 3:** Crude HTN prevalence in *All of Us* research program with age distribution in *All of Us* by gender.

Age Group	N Female (N = 19,704)	Female prevalence, % (95% CI)	N Male (N = 12,805)	Male prevalence, % (95% CI)	P-value
18–39	1351	7.1 (6.7–7.4)	710	9.2 (8.5–9.8)	< .0001
40–59	7280	31.0 (30.4–31.6)	4330	33.6 (32.8–34.5)	< .0001
≥ 60	11,073	49.2 (48.6–49.9)	7765	52.1 (51.3–52.9)	< .0001

.

**Figure 1:**
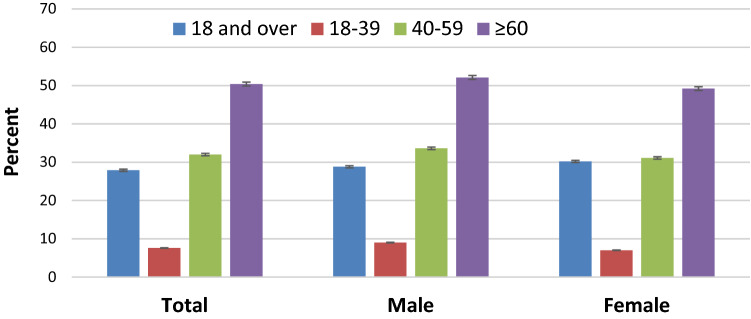
Prevalence of HTN among adults aged 18 and over, by age: *All of Us*, 2018–2019; ages 18 and over (blue), 18 to 39 (red), 40 to 59 (green), and 60 and over (purple) years. All estimates are age adjusted using the census population size at each age group as of July 1, 2018, based on https://www.census.gov/newsroom/press-kits/2019/detailed-estimates.html. Error bars show 95% confidence intervals for HTN prevalence estimates. Figure was created with Microsoft Excel for Mac, Version 16.46.

**Figure 2:**
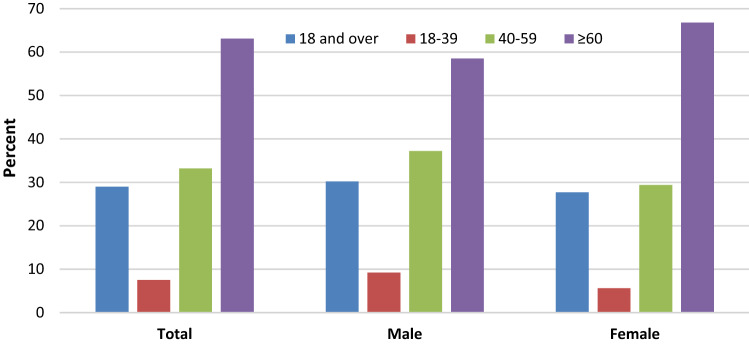
Prevalence of HTN among adults aged 18 and over, by age: United States, 2015–2016; ages 18 and over (blue), 18 to 39 (red), 40 to 59 (green), and 60 and over (purple) years. All estimates are age adjusted by the direct method using computed weights based on the subpopulation of persons with HTN in the 2007–2008 National Health and Nutrition Examination Survey, using age groups 18–39, 40–59, and 60 and over. Access data table for Fig. 2 at: https://www.cdc.gov/nchs/data/databriefs/db289_table.pdf#4. SOURCE: NCHS, National Health and Nutrition Examination Survey, 2015–2016. Figure was created with Microsoft Excel for Mac, Version 16.46.

*All of Us* data are skewed towards older age groups. Using methods of Crim, et. al.^7^ we calculated age-adjusted HTN prevalence based on the 2018 U.S. data. Age-adjusted HTN prevalence was 27.8% using 3 groups, 28.2% using 4 groups, and 28.5% using 5 groups. In comparison, NHANES age-adjusted prevalence was 29.6% for 3 groups, and 29.8% for 4 groups in NHANES 2007–2008 in Crim et al.^7^ Fig. 1 displays the prevalence of HTN calculated using *AoU* data (Fig. 1) and data from NHANES 2015–2016^9^ (Fig. 2).

Both figures show HTN prevalence in the 3 age groups (red, green and purple bars) and the overall age-adjusted prevalence (blue bar). Stratified by sex, age-adjusted prevalence (95% CI) was 28.7% (28.7–28.8) in males, 27.6% (27.57–27.58) in females in *AoU* vs. 30.2% in males and 27.7% in females in NHANES^9^. Table 4 shows the crude and age-adjusted HTN prevalence among race categories (as defined in US Census data), where American Indian and Alaska Native, and Native Hawaiian and Other Pacific Islander are combined as ‘Other’.

**Table 4:** HTN prevalence in the *All of Us* **R**esearch **P**rogram among race/ethnic groups adjusted for age based on U.S. Census data for age distribution of the population in 4 groups, 18–39, 40–59, 60–74, ≥ 75.

	All of Us research program		NHANES^1^
	Crude prevalence% (95% CI)	Age adjusted prevalence% (95% CI)	Age adjusted prevalence% (95% CI)
**Race**			
Black	43.0(42.4–43.7)	36.0(36.0–36.0)	40.3 (36.4–44.2)
White	30.6(30.2–31.0)	24.9(24.9–24.9)	27.8 (24.0–31.1)
Asian	20.5(19.0–22.0)	20.2 (20.1–20.2)	25.0 (21.7–28.3)
Mixed race	22.9(20.9–24.8)	19.0(18.9–19.0)	n/a
Other	28.1(27.5–28.7)	24.4(24.3–24.4)	n/a
**Ethnicity**			
Hispanic	25.6 (25.0–26.2)	21.1(21.1–21.1)	27.8 (24.0–31.1)
Non-Hispanic	31.3 (31.0–31.7)	26. 2 (26.2–26.2)	n/a

^1^All estimates are age adjusted by the direct method using computed weights based on the subpopulation of persons with HTN in the 2007–2008 National Health and Nutrition Examination Survey, using age groups 18–39, 40–59, and 60 and over. Access data table for Fig. 4 at: https://www.cdc.gov/nchs/data/databriefs/db289_table.pdf#4

SOURCE: NCHS, National Health and Nutrition Examination Survey, 2015–2016.

**Figure 3:**
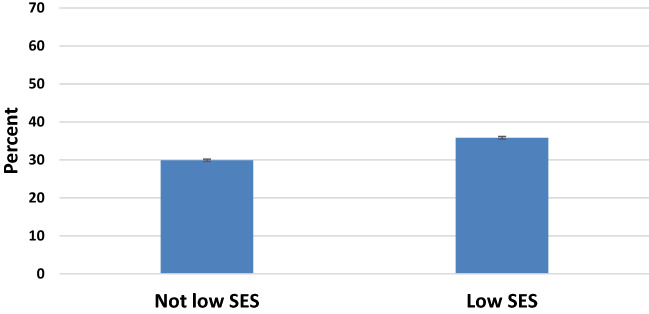
Crude HTN prevalence by SES in *All of Us,* 2018–2019. Error bars show 95% confidence intervals for HTN prevalence estimates. U.S. Census data is not available for age-distribution by SES categories. Figure was created with Microsoft Excel for Mac, Version 16.46.

Figure 3 shows crude HTN prevalence by socioeconomic status (SES) in *AoU,* 2018–2019. U.S. Census data is not available for age-distribution by SES categories. With respect to missing data, we noted that 28.1% (n = 29,024) did not report income and 2.2% (n = 2,312) did not report education. HTN prevalence (95% CI) stratified by income < 25,000 versus > 25,000 was 39.9% (39.1%–40.7) versus 30.4% (30.1–30.8), respectively. For individuals that did not report income, HTN prevalence was 37.0% (35.0–38.9). HTN prevalence (95% CI) stratified by education < high school/GED versus > one or more years of college was 34.9% (34.-35.4) versus 30.8% (30.4–31.1), respectively. For individuals that did not report education, HTN prevalence was 37.0% (35.0–38.9).

**Figure 4:**
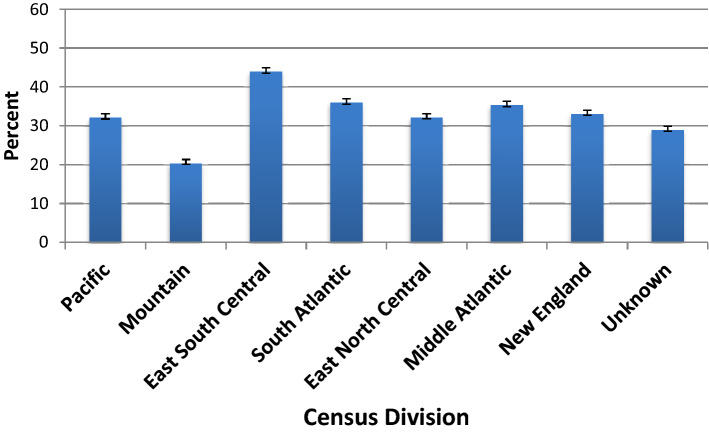
Crude HTN prevalence in *All of Us* by geographic region, 2018–2019. Error bars show 95% confidence intervals for HTN prevalence estimates. U.S. Census data is not available for age-distribution by geographic region. Figure was created with Microsoft Excel for Mac, Version 16.46.

Figure 4 shows crude HTN prevalence in *All of Us* by geographic region, 2018–2019.

U.S. Census data is not available for age-distribution by geographic region. HTN prevalence was higher among those who live in the Middle Atlantic, South Atlantic, and East South Central regions of the U.S. Prevalence was lower among those who live in the Mountain region of the U.S.

## Discussion

We completed the first analysis of HTN using data from the *AoU* Research Program Researcher Workbench. We reproduced known associations between race, SES, and geographic region and HTN^9^. The prevalence of HTN varies in the United States (U.S.) by age, sex, and socioeconomic status^9, 16^. *AoU* age-adjusted HTN prevalence using three age groups was 27.9% compared to 29.6% in NHANES. Using four age groups, aged-adjustment prevalence was 28.2% in *AoU* compared to 29.8%^7^. Fryar studied temporal trends in age-adjusted NHANES HTN prevalence, age-adjusted to four groups, in two year periods (2009–2016) with relatively stable rates of 28.6%, 28.7%, 29.3%, and 29.0% for 2015–2016^9^. Thus, *AoU* HTN prevalence is about 1% lower than reported prevalence in NHANES^9^. NHANES is considered a primary source of HTN statistics (e.g. prevalence and control) that informs public health and clinical care. We have shown that *AoU* data provides very similar prevalence estimates, which supports the data’s validity.

For more than 15 years, the U.S. saw a rise in blood-pressure (BP) control from 31.8% to 53.8%^17^. However, BP control dropped to 43.7% from 2013–2014 to 2017–2018^17^. A greater proportion of Americans, particularly those in marginalized communities, are living with uncontrolled HTN^18–20^. The drop in BP control highlights the need for healthcare providers to recommit to prioritizing BP control. Evidence suggests that computerized clinical decision support systems may be a promising tool for reducing the burden of HTN^6,21,22^. *AoU* may serve as a strategic platform to develop diversity-by-design rule-based algorithms for treatment and prevention of HTN that are generalizable to various populations. Researchers, clinicians, patients, and community stakeholders, and analytics professionals (and possibly more) are all needed to ensure that the right additional checks and balances are in place for responsible algorithm deployment. The *AoU* data is available to everyone. The open-access nature of *AoU* data may address inherent bias problems caused by the underrepresentation of diversity in the individuals that have access to data.

NHANES, another open-access cohort, captures data on a nationally-representative sample of approximately 5,000 participants annually. NHANES includes data from survey interviews and in-person physical measurements. NHANES defined HTN for participants by (a) systolic blood pressure ≥ 140 or diastolic blood pressure ≥ 90 mm Hg, (b) if the subject said “yes” to taking antihypertensive medication, or (c) if the subject was told on two occasions that the subject had HTN. For *AoU* data, we chose an EHR-based definition of hypertension^23–25^ instead of using a clinical definition such as the ACC/AHA Guidelines published in 2017^26^. Once the clinical diagnosis of HTN is made, clinicians and insurers make decisions using the EHR-based definition^27^. Thus, our EHR-based HTN findings that replicate NHANES’ HTN prevalence^9^ have important real-world implications for improving the management of HTN.

We demonstrated some modest differences in sex stratified HTN prevalence: age-adjusted male prevalence was 28.8% in *AoU* compared to 30.2% in NHANES and age-adjusted female prevalence was 30.2% in *AoU* vs. 27.7% in NHANES^9^. These differences could be due to inclusion of HTN medication use in our HTN definition. In prior work, Geldsetzer, et al. reported that among those with HTN, 39.2% were aware of their diagnosis, 29.9% had received treatment, and 10.3% had control of their HTN^28^. They also reported that older age, female or a non-smoker, and higher levels of education and income were associated with higher progression through the cascade of HTN care^28^. HTN can often be treated successfully with medication^29–32^ and prevented or delayed with lifestyle modifications^32–34^. Even with these established HTN intervention and prevention strategies, the prevalence of HTN continues to be at levels of public health concern^1^.

## Limitations

EHRs were limited to data that is collected within a single healthcare network, and thus may not capture out of network care. In theory, *AoU* will ultimately include EHR data from individuals across multiple institutions. Some *AoU* recruitment sites are in the process of EHR data extraction and transfer to the Data Research Center. We currently do not have information on data completeness from each recruitment site in the *AoU* Research Program. Thus, our preliminary findings may underestimate HTN prevalence in the U.S. The geographic representation in the *AoU* Research Program is currently weighted towards regions with healthcare provider organizations that are funded for large scale recruitment. As more direct volunteers are recruited in the future, we expect the geographic representation to improve.

## Strengths

The *AoU* dataset provides advantages over datasets like NHANES. *AoU* has more covariates such as EHR data and genetic information for broader analyses. Data from *AoU* may contribute additional value to existing national resources used to study HTN through the scale at which measured data are available. Using the entire EHR allowed us to extract coded data on HTN diagnoses and medications, a method that has been shown to be valid by the eMERGE consortium^15^. To avoid a racially biased algorithm^35^, the diagnostic algorithm for hypertension did not use race or ethnicity data. Additionally, the diversity within *AoU* may provide insight into factors relevant to HTN prevention and treatments in a variety of social and geographic contexts and population strata in the U.S. given that over 80% of *AoU* participants have been historically underrepresented in biomedical research from the perspectives of age, race/ethnicity, sexual orientation and gender identity, geography or other dimensions.

In summary, the *AoU* Research Program data capture known differences in the prevalence of HTN by demographic^7^ and geographic characteristics. *AoU* has great potential to contribute to the vision of precision medicine for hypertension to improve clinical outcomes in patients with and at-risk for HTN. Future research that takes advantage of the rich data (including social determinants of health, genomics and biomarkers) in *AoU* may lead to novel insights into differences among under-represented groups. This cohort presents the opportunity to analyze data streams derived from genomics combined with clinical and geographical data to discover mechanisms and potential target molecules from which drugs or treatments can be developed.

## Data Availability

Access to the Researcher Workbench and data is free. All researchers must be authorized and approved via a 3-step process that includes registration, completion of ethics training and attestation to a data use agreement.
